# Mac-2-binding protein glycan isomer enhances the aggressiveness of hepatocellular carcinoma by activating mTOR signaling

**DOI:** 10.1038/s41416-020-0971-y

**Published:** 2020-07-06

**Authors:** Gantumur Dolgormaa, Norifumi Harimoto, Norihiro Ishii, Takahiro Yamanaka, Kei Hagiwara, Mariko Tsukagoshi, Takamichi Igarashi, Akira Watanabe, Norio Kubo, Kenichiro Araki, Tadashi Handa, Takehiko Yokobori, Tetsunari Oyama, Hiroyuki Kuwano, Ken Shirabe

**Affiliations:** 1 Department of Hepatobiliary and Pancreatic Surgery, Gunma University, Graduate School of Medicine, 3-39-22 Showamachi, Maebashi, 371-8511 Gunma Japan; 2 Department of General Surgical Science, Gunma University, Graduate School of Medicine, Medicine, 3-39-22 Showamachi, Maebashi, 371-8511 Gunma Japan; 3 Department of Innovative Cancer Immunotherapy, Gunma University, Medicine, 3-39-22 Showamachi, Maebashi, 371-8511 Gunma Japan; 4 Department of Diagnostic Pathology, Gunma University, Graduate School of Medicine, Medicine, 3-39-22 Showamachi, Maebashi, 371-8511 Gunma Japan; 5 Department of Social Welfare, Gunma University of Health and Welfare, 191-1 Kawamagarimachi, Maebashi, Gunma Japan; 6 Gunma University Initiative for Advanced Research (GIAR), Medicine, 3-39-22 Showamachi, Maebashi, 371-8511 Gunma Japan

**Keywords:** Hepatocellular carcinoma, Hepatocellular carcinoma

## Abstract

**Background:**

Wisteria floribunda agglutinin (WFA)^+^ Mac-2-binding protein (M2BPGi) is a novel serum marker for liver fibrosis. Although an elevated serum level of M2BPGi can predict development of hepatocellular carcinoma (HCC), the effect of M2BPGi on HCC remains unclear. There are no reports about the association of M2BPGi with HCC aggressiveness. We aimed to clarify the significance of M2BPGi in HCC.

**Methods:**

The protein expression of M2BPGi and galectin-3, a ligand of M2BP, and the mRNA expression of *M2BP* were evaluated in surgically resected human HCC samples. M2BPGi-regulating signals in HCC cells were investigated using transcriptome analysis. The effects of M2BPGi on HCC properties and galectin-3/mTOR signaling were evaluated.

**Results:**

M2BPGi and galectin-3 proteins co-localised in HCC cells, while *M2BP* mRNA was detected in cirrhotic liver stromal cells. mTOR signaling was upregulated in M2BPGi-treated HCC cells. Moreover, M2BPGi treatment induced tumour-promoting effects on HCC in vitro by activated mTOR signaling. In addition, M2BPGi bound to galectin-3 to induce membranous galectin-3 expression in HCC cells. In vivo, M2BPGi enhanced the growth of xenografted HCC.

**Conclusions:**

M2BPGi is produced in stromal cells of the cirrhotic liver. Furthermore, M2BPGi enhances the progression of HCC through the galectin-3/mTOR pathway.

## Background

Liver cancer is the sixth and ninth most common type of cancer in men and women, respectively, and the fourth most common cause of cancer-related death worldwide. The majority of liver cancers involve the histological subtype hepatocellular carcinoma (HCC), a primary malignancy of the liver.^1^ Several liver factors, particularly fibrosis, contribute to the risk of hepatocarcinogenesis. Approximately 70–90% of patients with HCC have a history of chronic liver disease and cirrhosis,^2^ and patients with liver cirrhosis (Child–Pugh score B or C) who develop HCC have a significantly worse prognosis than those without cirrhosis.^3^ Although liver resection is the most effective treatment for patients with early HCC who have a non-cirrhotic or mildly cirrhotic liver,^4^ few treatment options are available for those with severe liver fibrosis.^5^ Therefore, a deeper understanding of HCC in fibrotic liver is essential for its clinical management.

According to Kuno et al., Wisteria floribunda agglutinin (WFA) lectin can bind to terminal N-acetylgalactosamine motifs on carbohydrate structures, as well as with Mac-2-binding protein (M2BP, also known as galectin-3-binding protein) glycoforms during liver fibrosis.^6^ Accordingly, WFA-binding M2BP has been termed WFA^+^ M2BP or Mac-2-binding protein glycan isomer (M2BPGi).^6^ Clinical studies have identified M2BPGi as a novel serum marker of liver fibrosis associated with various aetiologies, including hepatitis B and C virus infections, non-alcoholic fatty liver disease and primary biliary cirrhosis,^7–11^ and the serum M2BPGi level may predict HCC development and prognosis in patients with hepatitis B and C virus infections.^12–15^ Bekki et al. reported that M2BPGi is secreted by hepatic stellate cells (HSCs) in the cirrhotic liver and subsequently activates Kupffer cells to promote hepatic fibrosis.^16^ Although a few studies addressed the source of M2BPGi in the cirrhotic liver, no basic studies have explained the potential association of M2BPGi with HCC aggressiveness.

mTOR signaling is upregulated in 40–50% of HCCs,^17^ and this pathway has been studied for the development of molecular targeted therapies for several cancers, including HCC.^18^ Interestingly, mTOR signaling is regulated by galectin-3,^17,18^ a well-known binding partner of M2BP protein. Although the expression of galectin-3 was reported to be higher in HCC than non-cancerous tissues, and to associate with a poor prognosis,^19–21^ the relationship between mTOR signaling, galectin-3 and M2BPGi in HCC remains unknown.

The investigations in the present study were conducted to clarify the source of M2BPGi in the liver, and the fundamental effects of M2BPGi against HCC cells. Specifically, the localisation of glycosylated M2BP as M2BPGi, galectin-3 and *M2BP* mRNA was evaluated in HCC tissues resected from fibrotic liver, and the tumour-promoting effects of M2BPGi were analysed in vitro and in vivo. Furthermore, Cap Analysis of Gene Expression (CAGE) was used to identify the relationship between M2BPGi and the novel downstream factors of galectin-3 and mTOR signaling in HCC cells.

## Methods

### Multicolour immunofluorescence staining

Five surgically resected HCC specimens were fixed with 1% formaldehyde, embedded in paraffin, cut into 4-µm-thick sections and mounted on glass slides. Deparaffinisation, antigen retrieval, endogenous peroxidase blocking and blocking protocols were described previously.^22^ The sections were incubated with primary antibodies, including rat anti-galectin-3 antibody (Cedarlane), goat anti-M2BP antibody (R&D Systems) and biotinylated WFA lectin (Vector Laboratories), and the appropriate Alexa Fluor-conjugated secondary antibodies and fluorescent streptavidin conjugates (1:1000, Invitrogen) for 60 min per incubation. The cell nuclei were labelled with Spectral DAPI (PerkinElmer). The labelled cells were visualised using a BZ-X710 fluorescent microscope (Keyence).

### RNA in situ hybridisation assay

Three paraffin-embedded tissue sections on slides were subjected to RNA in situ hybridisation to detect the localisation and expression of *M2BP* RNA, using a *M2BP*-specific probe and RNAscopeR2.5 HD Detection kit (BROWN; ACD Bio-Techne). The technique was performed manually according to the manufacturer’s protocol. The resulting images were evaluated using a light microscope (Carl Zeiss Axio Scope.A1 microscope).

### Cell culture

To validate the importance and function of M2BPGi in several differentiated cell lines of HCC, we selected the PLC/PRF/5 cell line to represent poorly differentiated HCC,^23^ whereas other cell lines (Huh7 and HepG2) represented well-differentiated HCC.^24^ All cell lines were obtained from the JCRB Cell Bank (Osaka, Japan). The cells were cultured in Dulbecco’s modified Eagle’s medium (DMEM) containing L-glutamine, Phenol Red and Sodium Pyruvate (Fujifilm Wako Pure Chemical Corporation) and supplemented with 10% foetal bovine serum (FBS) and 1% penicillin–streptomycin (ThermoFisher Scientific), and were incubated at 37 °C in a humidified incubator at 5% CO_2_.

### Proliferation assay

Cell-proliferation ability was analysed using Cell counting kit-8 (CCK-8, Dojindo Laboratories, Kumamoto, Japan). HCC cells were seeded at a density of 3000 cells/well in 96-well culture plates. After 24 h, the medium was changed with and without 1 or 3 µg/ml M2BPGi (Sysmex Co.), sugar chain cut-off—M2BPGi (SC-M2BPGi) and 10 nM mTOR inhibitor (rapamycin, EMD Millipore). Cell proliferation was evaluated at initial seeding (0 h) and at 24, 48 and 72 h of treatment. The absorbance of each well was measured using a spectrophotometer (Bio-Rad, Hercules, CA, USA) at 450 nm with the reference wavelength set at 650 nm. All experiments were performed in replicates of six.

### Invasion assay

The cell-invasion assay was performed using 24-well Corning^®^ BioCoat™ Matrigel Invasion Chambers (Corning, NY, USA). HCC cell lines in FBS-free medium were seeded into the Matrigel inserts at a density of 1 × 10^5^ per chamber. The lower chamber contained 1 or 3 µg/ml M2BPGi in medium containing 10% FBS. After incubation for 48 h, the cells were fixed and stained with Diff-Quik (Sysmex Corporation, Kobe, Japan). After staining, cells that migrated through the pores to the lower surface of the membrane were counted under the microscope. In total, five randomly selected fields were evaluated.

### Wound-healing assay

Cell-migration ability was evaluated in M2BPGi non-treated and treated HCC cells (PLC/PRF/5) using wound-healing assay. The cells were seeded in the six-well plates and cultured until reaching confluence. A uniform straight wound was generated using pipette tip. Each well was washed with PBS to remove detached cells, and wells were cultured in medium with and without M2BPGi (3 µg/ml) at 37 °C for 48 h. The cell-migration rate was calculated using replicates of five.

### In vivo experiment

Six female NOD-SCID mice (7 weeks old, body weight: 19–20 g) were purchased from CLEA Japan, Inc. Mice have free access to water and food, housed in specific pathogen-free cages and bedding in a 12-h light/dark regimen with controlled room temperature. PLC/PRF/5 cells were suspended in 10% FBS DMEM at a density of 5 × 10^6^/200 µl and injected subcutaneously into both flanks of each mouse. Each group contained five xenografts. After 7 days, the mice were randomly divided into control (PBS) and M2BPGi groups and implanted subcutaneously with mini-osmotic pumps (Alzet^®^, model 2002) charged with PBS or M2BPGi (303 µg/ml), respectively. M2BPGi was secreted subcutaneously with 3.6 µg/ml a day for 14 days. During the procedures of injection and pump implantation, mice were anaesthetised with isoflurane (Pfizer Japan Inc., Tokyo). The mice were observed until complete recovery from anaesthesia. The tumour sizes and body weights were measured every 2 days after pump implantation, and the tumour volumes were calculated using the following formula: V = (W^2^ × L)/2. To collect the xenografted tumours, mice were deeply anaesthetised with isoflurane and euthanised by cervical dislocation on the 14th day of M2BPGi administration. The resected tumours were fixed with formalin-neutral buffer 20 (Kenei Pharmaceutical, Japan) for pathological examination. All mouse experiments were conducted in compliance with the guidelines of the Institute for Laboratory Animal Research at Gunma University, Maebashi, Japan (Approval number: 18-024).

### Immunohistochemistry

Immunohistochemical staining was performed using antibodies specific for Ki-67 (Dako, Agilent Technologies) and phospho-S6 (anti-rabbit, Cell Signaling Technology) according to previously described methods.^22^ Ki-67-positive cancer cells from samples treated with PBS and M2BPGi were counted directly on 10 representative images at the same magnification (×400).

### siRNA transfection

HCC cells were suspended in Opti-MEM I-reduced Serum Media (ThermoFisher Scientific) at a density of 1.0 × 10^6^/100 µl and mixed with control siRNA (Dharmacon) or the following galectin-3 siRNAs at a concentration of 200 µM: #1, sense: 5′-GGAGAGUCAUUGUUUGCAA-3′, antisense: 5′-UUGCAAACAAUGACUCUCC-3′; siRNA #2, sense: 5′-GGGAAUGAUGUUGCCUUCCACUUUA-3′, antisense: 5′-UAAAGUGGAAGGCAACAUCAUUCCC-3′. All siRNA transfections were performed via electroporation using a CUY21 EDIT II electroporator (BEX) at poring and transfer pulses of 150 and 10 V, respectively.

### Protein extraction and Western blotting analysis

HCC cell lines were treated with M2BPGi at concentrations varying between 0 µg/ml and 3 µg/ml for 6 h. After 24 h of galectin-3 siRNA transfection, cells were treated with 1 µg/ml of M2BPGi for 24 h, followed by protein extraction. Protein extraction was performed using RIPA buffer (Wako) containing phosphatase inhibitor (p2850, Sigma-Aldrich) according to the manufacturer’s instructions.

Membrane proteins were extracted using a Mem-PER Plus Membrane protein extraction kit (ThermoFisher Scientific) according to the manufacturer’s protocol. An approximately 2-mm section of xenograft tissue was homogenised in RIPA buffer (Wako) containing phosphatase inhibitor (p285, Sigma-Aldrich) according to the manufacturer’s protocol.

Proteins were separated using SDS-PAGE with 10% Bis–Tris gels and transferred to nitrocellulose membranes (#12369, Cell Signaling). Membranes were blocked with 5% skimmed milk or 5% BSA and incubated overnight at 4 °C with the primary antibodies. Primary antibodies used in the present study were anti-galectin-3 (Cedarlane), phospho-mTOR, mTOR, phospho-p70–S6K, p70–S6K, phospho-S6 (Cell Signaling Technology) and beta-actin (Sigma). Next, membranes were treated with horseradish peroxidase (HRP)-conjugated secondary antibodies. Protein bands on the membrane were detected using ECL™ Prime Western Blotting Detection Reagent and an ImageQuant™ LAS 4000 imager (GE Healthcare, Buckinghamshire, UK).

### Co-immunoprecipitation

PLC/PRF/5 cells were incubated with or without 3 µg/ml M2BPGi for 12 h and subsequently collected and lysed with 1% Triton-X. For co-immunoprecipitation, a lysate volume containing 300 µg of protein was incubated with 3 µl of human galectin-3 antibody (R&D Systems) for 2 h at 4 °C with constant rotation. Subsequently, 20 µl of protein G beads were added, and the mixture was incubated for another 1 h with constant rotation. After 5 washes, the precipitated proteins were resuspended in 20 µl of SDS sample buffer and boiled at 95 °C for 5 min. Protein expression was evaluated by western blotting.

### Cap analysis of gene expression

PLC/PRF/5 cells were seeded in 10-cm dishes and grown to 70–80% confluency at 37 °C in a humidified incubator with 5% CO_2_. The medium was supplemented or not with 3 µg/ml M2BPGi. After 6 h, the cells were collected and washed with PBS, snap-frozen in nitrogen and stored at −80 °C. The CAGE library preparation, sequencing, mapping and gene expression and motif discovery analysis were performed by DNAFORM. Briefly, RNA quality was assessed using a Bioanalyzer (Agilent) to ensure that the RNA-integrity number (RIN) exceeded 7.0, and the A260/280 and A260/230 ratios exceeded 1.7. First-strand cDNAs were transcribed to the 5′ ends of capped RNAs and attached to CAGE ‘bar code' tags. The sequenced CAGE tags were then mapped to mouse mm9 genomes using BWA software (v0.5.9) after discarding ribosomal or non-A/C/G/T base-containing RNAs. Finally, the CAGE-tag 5′ coordinates were input for CAGEr clustering^25^ using the Paraclu algorithm^26^ with the default parameters.

### Statistical analysis

The experimental data of the proliferation, invasion and xenografted tumour-volume assays were analysed using replicates of 5–6. Data for continuous variables are expressed as means ± standard deviations (SDs). Differences among groups were evaluated using the *t* test and Tukey’s test. In vivo data were analysed using repeated-measures analysis of variance. The results with *P* values < 0.05 were considered statistically significant. All statistical analyses were conducted using the JMP 13.0.0 software package (SAS Institute Inc.).

## Results

### Co-localisation of M2BPGi and galectin-3

**Fig. 1:**
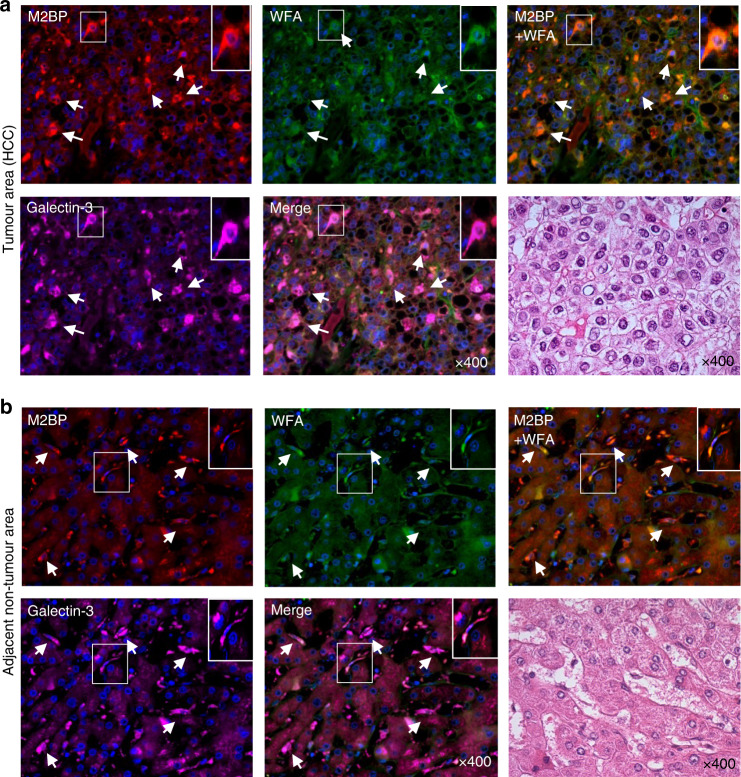
Co-localisation of M2BPGi and galectin-3 in HCC and Kupffer cells. Representative multi-immunofluorescence images of cells labelled to indicate M2BP (red, upper left panel), WFA (green, upper middle panel), M2BP with WFA as M2BPGi (merged, upper right panel), galectin-3 (pink, lower right panel) or M2BPGi and galectin-3 (merged, lower middle panel) and stained with haematoxylin and eosin (lower right panel) in the tumour (**a**) and adjacent non-tumour (**b**) areas of resected hepatocellular carcinoma tissue (×400 magnification).

In a previous study, M2BPGi-positive cells were investigated in fibrotic liver samples using immunohistochemical analysis.^16^ In our study, we evaluated M2BPGi expression in HCC tissue samples using immunohistochemistry. The results revealed that M2BPGi and galectin-3 were co-localised in HCC cells (Fig. 1a). In addition, adjacent non-tumour areas were analysed. M2BPGi co-localised with a Kupffer cell marker (CD68) (Supplementary Fig. 1) and galectin-3 (Fig. 1b) according to immunofluorescence staining.

### Expression of *M2BP* mRNA

**Fig. 2:**
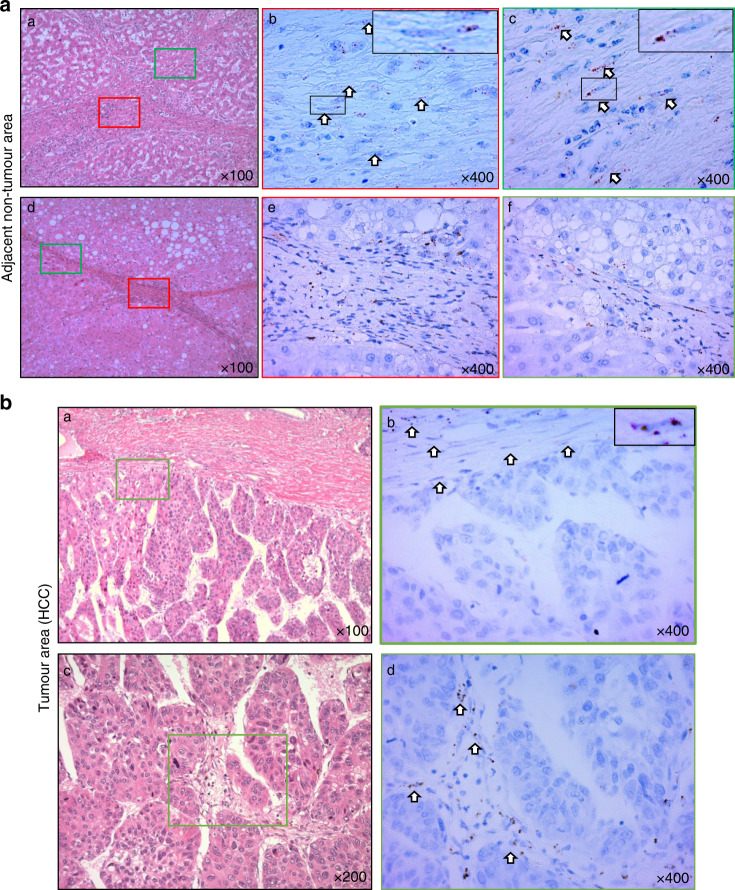
M2BPGi is derived from stromal cells of the cirrhotic liver. **a** The expression of *M2BP* mRNA was assessed using RNA in situ hybridisation. *M2BP* mRNA was detected (arrows) in stromal cells of the cirrhotic liver (b, c) but not in hepatocytes (e, f) at ×400 magnification. Haematoxylin–eosin staining (a, d) at ×100 magnification. **b** *M2BP* mRNA was detected (arrows) in stromal cells of the HCC but not in HCC cells (b, d) at ×400 magnification. Haematoxylin–eosin staining (a, c) at ×100 and ×200 magnification.

In addition, to investigate the source of M2BP protein, we examined *M2BP mRNA* expression using an in situ hybridisation assay. *M2BP* mRNA expression was detected in the stroma of the cirrhotic liver rather than in hepatocytes (Fig. 2a). In addition, we analysed *M2BP* mRNA expression in HCC tissue samples. *M2BP* mRNA expression was detected in the stroma of HCC tissues, but not in HCC cells (Fig. 2b). According to the result, we examined M2BP protein expression in HCC cell lines, including PLC/PRF/5, HepG2 and Huh7 cells. M2BP was expressed in PLC/PRF/5 and HepG2 cells, but not in Huh7 cells (Supplementary Fig. 2).

### M2BPGi treatment and HCC progression

**Fig. 3:**
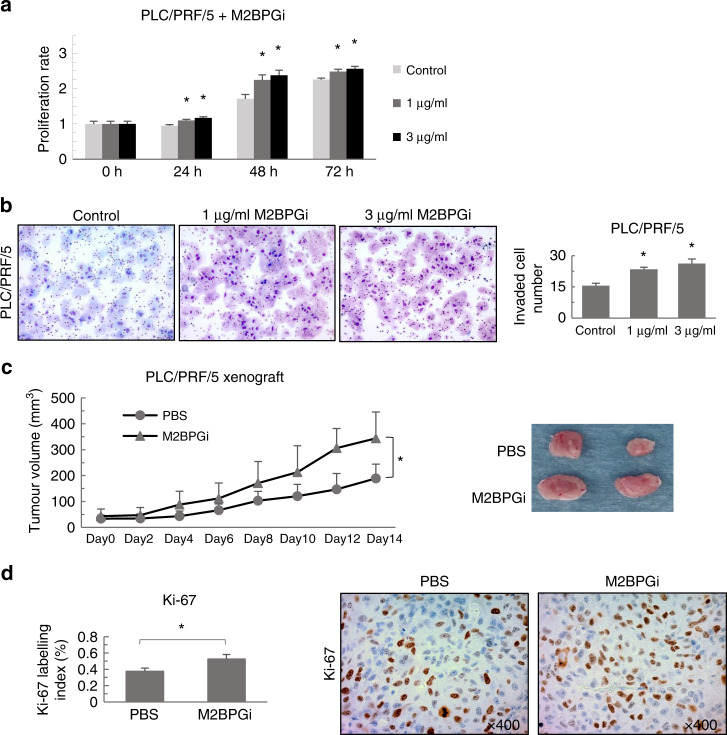
M2BPGi treatment enhanced the progression of HCC in vitro and in vivo. HCC cell lines (PLC/PRF/5) treated with M2BPGi were subjected to **a** a proliferation assay (*n* = 6, *p < 0.05) and **b** invasion assay (*n* = 5, *p < 0.05). **c** M2BPGi significantly enhanced the growth of xenografted tumours when compared with controls (PBS) (*n* = 5, *p = 0.0023). **d** The Ki-67 labelling index was significantly higher in M2BPGi-treated xenograft tumours than in controls (*n* = 10, *p < 0.01) (×400 magnification).

M2BPGi significantly enhanced the proliferation rates of PLC/PRF/5 human HCC cell lines when compared with non-treated cells (Fig. 3a). Other HCC cell lines (HepG2 and Huh7) showed the same result (Supplementary Fig. 3a). On the other hand, sugar chain cut-off M2BPGi (SC-M2BPGi) had no proliferation effect on PLC/PRF/5 cell line (Supplementary Fig. 4). M2BPGi also significantly enhanced the invasiveness and migration of the PLC/PRF/5 cell line when compared with non-treated cells (Fig. 3b) (Supplementary Fig. 3c). HepG2 and Huh7 cell lines showed similar results with PLC/PRF/5 cell line (Supplementary Fig. 3b). In vivo, we confirmed that treatment with M2BPGi enhanced tumour growth significantly in an HCC-bearing mouse model, compared with controls (Fig. 3c). M2BPGi supported the proliferation of HCC cell lines in vitro; therefore, we analysed the expression of the proliferation marker Ki-67 in the xenografted tumours. The Ki-67 labelling index was higher in M2BPGi-treated than in PBS-treated tumours (Fig. 3d).

### Membranous galectin-3 and tumour-promoting effect of M2BPGi

**Fig. 4:**
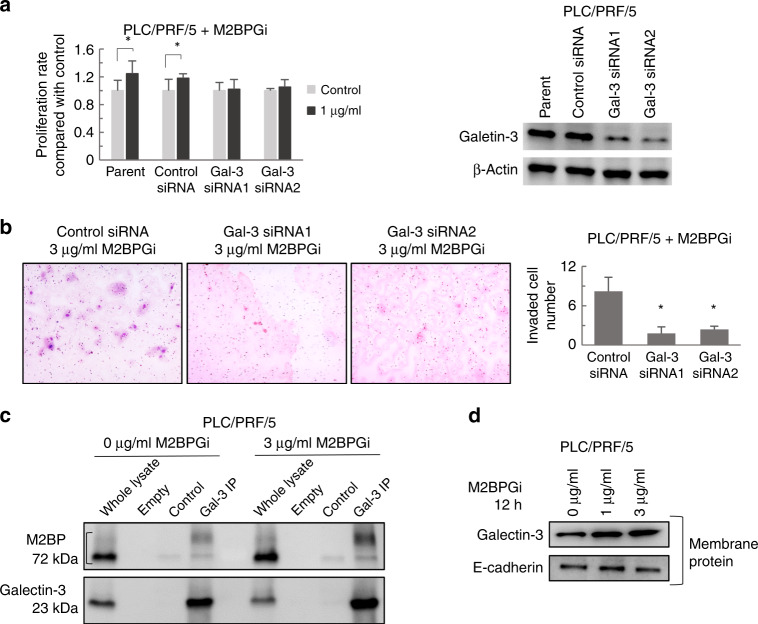
M2BPGi promoted HCC tumorigenesis through interactions with galectin-3 (Gal-3). **a** Compared with control, M2BPGi did not enhance the proliferation of HCC cell lines (PLC/PRF/5) transfected with Gal-3 siRNA (*n* = 6, *p < 0.01). Western blotting validated the suppression of Gal-3 by specific siRNAs. **b** M2BPGi did not enhance the invasion of HCC cell lines (PLC/PRF/5) transfected with Gal-3 siRNA compared with controls (*n* = 5, *p < 0.01). **c** M2BP immunoprecipitation using a Gal-3-specific antibody (Gal-3 IP)-detected M2BP at 75-kDa band in whole-cell lysate. M2BPGi-treated Gal-3 IP samples, but not controls, appeared as a broad M2BP band at 85–97 kDa. **d** Membranous Gal-3 protein expression in PLC/PRF/5 cells treated with M2BPGi. E cadherin was used as a membrane protein- loading control.

Next, we focused on the relationship between M2BPGi as a tumour promoter and galectin-3 as a representative M2BP-binding protein. Notably, M2BPGi treatment did not enhance the proliferation of galectin-3–siRNA-knockdown HCC cells relative to controls (Fig. 4a) (Supplementary Fig. 5). Moreover, M2BPGi did not enhance the invasive ability of galectin-3-downregulated cells compared with the findings in the controls (Fig. 4b). To further evaluate the interaction of M2BPGi with galectin-3 in HCC cells, we subjected the PLC/PRF/5 HCC cell line to immunoprecipitation, and observed stronger M2BP expression (85–97 kDa) in M2BPGi-treated cells than in non-treated cells. Glycosylation at N-linked sites of M2BP generates molecular size of 85–97 kDa,^27^ which represents M2BPGi. Our result indicates that extrinsic M2BPGi could bind to cellular galectin-3 (Fig. 4c). Furthermore, the membranous expression of galectin-3 increased in response to M2BPGi treatment (Fig. 4d).

### M2BPGi and mTOR signaling in HCC cells

**Fig. 5:**
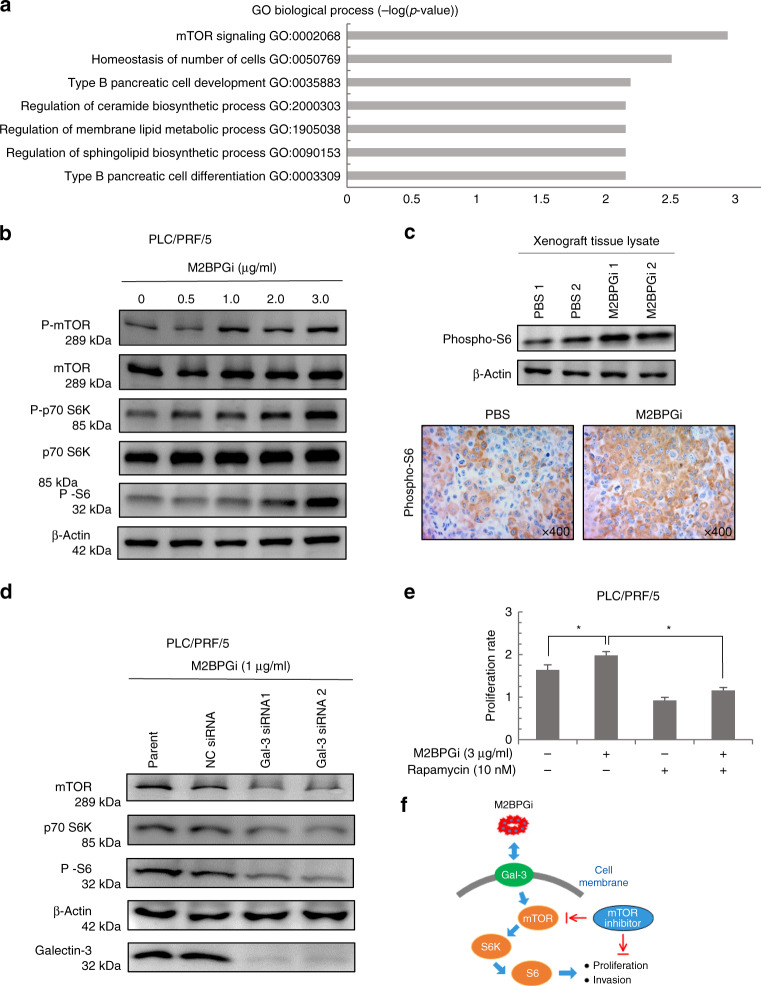
M2BPGi enhances mTOR signaling in HCC cells in the presence of galectin-3 (Gal-3). **a** Enhanced mTOR signaling in M2BPGi-treated PLC/PRF/5 cells by GO analyses. **b** M2BPGi induced the phosphorylation of mTOR, p70–S6K and S6 in PLC/PRF/5 cells. **c** Phospho-S6 was enhanced in the lysates of M2BPGi-treated xenograft tumour tissues. Representative images of phospho-S6 staining (×400 magnification). **d** Gal-3 downregulation reduced the expression of mTOR -signaling pathway. **e** The proliferative effects of M2BPGi on HCC cells were inhibited by treatment with rapamycin (mTOR inhibitor) (*n* = 6, *p < 0.01). **f** Schema of the mTOR-meditated functions of M2BPGi.

To clarify the mechanism by which M2BPGi promotes HCC, we subjected PLC/PRF/5 cells to a CAGE analysis. A gene ontology analysis suggested that mTOR signaling was upregulated in M2BPGi-treated cells relative to non-treated cells (Fig. 5a). Therefore, we evaluated the induction of mTOR, p70–S6 kinase and S6 as representative downstream components of the mTOR- signaling pathway in M2BPGi-treated cells. We further validated the dose-dependent induction of mTOR, p70–S6 kinase and S6 phosphorylation in M2BPGi-treated cells by western blotting (Fig. 5b).

Consistent with our in vitro data, we also observed enhanced levels of phospho-S6, an indicator of activated mTOR signaling, in the M2BPGi-treated xenograft tumours relative to the controls (Fig. 5c). We next evaluated whether galectin-3 was required for the activation of mTOR signaling in HCC cells treated with M2BPGi. We observed downregulated mTOR signaling in the galectin-3–siRNA-knockdown cells relative to the levels in control cells, regardless of the M2BPGi treatment status (Fig. 5d). Finally, although M2BPGi treatment enhanced the proliferation of HCC cells (Fig. 3a), the tumour-promoting effects of M2BPGi were cancelled by treatment with the representative mTOR inhibitor rapamycin (Fig. 5e).

## Discussion

In this study, we focused on the liver fibrosis marker, M2BPGi, a glycosylated galectin-3-binding protein derived from the stromal cells of cirrhotic liver. We further showed that M2BPGi could bind to and induce the membrane expression of galectin-3. Moreover, M2BPGi promoted HCC malignancy in vitro via galectin-3, which consequently activated mTOR signaling. This was confirmed by the observation that galectin-3 knockdown significantly inhibited the effect of M2BPGi on HCC cells. Our in vivo findings were consistent with the in vitro data and further demonstrated the importance of M2BPGi-induced mTOR signaling on HCC growth.

To date, to the best of our knowledge, no study has clarified the source of M2BPGi in fibrotic liver tissues. Accordingly, this report is the first to confirm a discrepancy between the localisation of M2BPGi protein and *M2BP* mRNA in clinical samples. In a previous study, an anti-M2BP antibody and WFA lectin were used to detect glycosylated M2BP protein (i.e. M2BPGi) via staining only in human tissue samples.^16^ The protein localisation data suggested the importance of M2BPGi in the fibrotic liver.^16^ In addition, they described the origin of M2BPGi in the fibrotic liver using HSCs, Kupffer cells, biliary epithelial cells and endothelial cells, which were isolated from human fibrotic liver tissues. The results illustrated that HSCs dominantly secreted M2BPGi, thereby activating Kupffer cells in vitro to facilitate liver fibrosis.^16^ Moreover, M2BPGi has been used as a clinical serum biomarker of liver fibrosis,^28^ a poor prognosticator in patients with HCC.^3,29^ Our immunohistochemical data revealed that M2BPGi expression was observed in HCC cells within the tumour areas, and within Kupffer cells rather than hepatocytes in the adjacent non-tumour areas. Therefore, to clarify the source of M2BPGi in the liver tissues, we identified the stromal cells of the cirrhotic liver, rather than the hepatocytes, as the M2BP-producing cells using RNA in situ hybridisation. Taken together, these data suggest that M2BPGi is secreted from stromal cells and may be taken up by HCC cells in the fibrotic liver.

Previously, M2BP protein was reported to affect cancer aggressiveness. In human cancers, M2BP was shown to bind to galectin-3,^30,31^ which was related to the activation of mTOR signaling in cancer cells.^17^ However, glycosylation of M2BP was not reported in previous studies. The suppression of galectin-3 downregulated the activation of mTOR and S6K in colon cancer.^18^ In this study, we used CAGE analysis to identify mTOR signaling as a downstream effector of M2BPGi in HCC, and validated that M2BPGi could bind to galectin-3 and activate mTOR signaling in HCC cells through both in vitro and in vivo experiments. Moreover, mTOR inhibitor treatment and galectin-3 suppression blocked the effects of M2BPGi on HCC aggressiveness. Therefore, our results suggested that M2BPGi may enhance HCC aggressiveness by activating the galectin-3/mTOR axis.

In this study, the activation of mTOR signaling by M2BPGi/galectin-3 was reported; however, the fundamental mechanism by which galectin-3 activates mTOR was not evaluated. In another study, galectin-3 was reported to activate MAPK signaling, which plays a key role in the progression of cancers, including HCC.^32,33^ Conversely, MAPK signaling has been reported to be upstream of mTOR in lung cancer cells.^34^ Taken together, these reports suggest that M2BPGi-induced galectin-3 enhances mTOR signaling through MAPK signaling. Although mTOR inhibitors have been developed and used in clinical trials for HCC treatment in the past decade, the tumour-suppressive effects of these agents in patients with HCC remain controversial.^35,36^ One randomised, multicenter, multinational Phase 2 trial reported that the addition of an mTOR inhibitor to sorafenib did not improve the clinical efficacy compared with sorafenib alone in patients with advanced HCC.^36^ Moreover, several researchers reported activated mTOR signaling in HCC,^37–39^ and clinical studies identified mTOR activation as a potential indicator of a poor prognosis in patients with HCC.^40^ In our study, we demonstrated that M2BPGi could enhance the aggressiveness of HCC by activating mTOR signaling in the presence of galectin-3. Our data suggest that the tumour expression of galectin-3 may be a predictive factor of treatment outcomes, and could be used to select patients who would benefit from mTOR inhibitor therapy. Our data further suggest that M2BPGi may regulate the magnitude of mTOR activation in HCC; in other words, the serum level of M2BPGi may reflect the level of mTOR activation in HCC cells. Potentially, M2BPGi may be a useful non-invasive marker for the identification of patients with HCC with mTOR activation. In the future, a therapeutic strategy based on mTOR inhibitors might be useful for the treatment of mTOR-activated HCC associated with a high level of M2BPGi.

In conclusion, M2BPGi is produced by stromal cells in the cirrhotic liver, where it enhances the progression of HCC by activating mTOR signaling in the presence of galectin-3. M2BPGi may serve as a link between HCC and stromal cells, and may thus explain the fundamental mechanism underlying HCC progression. Accordingly, M2BPGi may be a promising therapeutic target in patients with liver cirrhosis who develop HCC.

## Supplementary information

Supplementary Figures

Supplementary Figure legends

## Data Availability

All data generated or analysed during this study are included in this paper. CAGE data reported in this study have been deposited to the GEO (Gene Expression Omnibus) and are available through the accession number [GSE134471].
