# Supplementary material for: Mac-2-binding protein glycan isomer enhances the aggressiveness of hepatocellular carcinoma by activating mTOR signaling
Source: Br J Cancer. 2020 Jul 6;123(7):1145–53. doi: 10.1038/s41416-020-0971-y (PMC7525442; doi:10.1038/s41416-020-0971-y)

Supplementary Figure 1

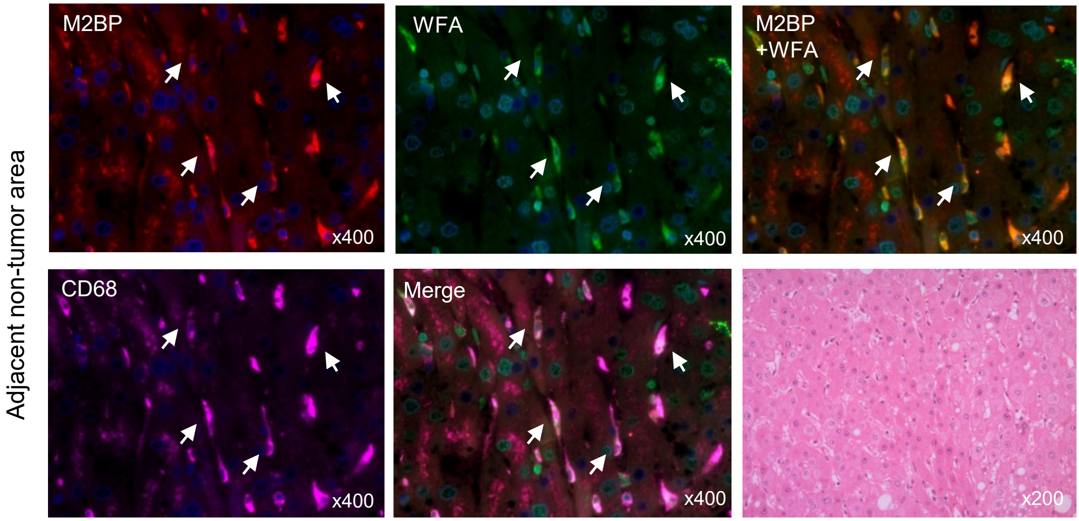

Supplementary Figure 2

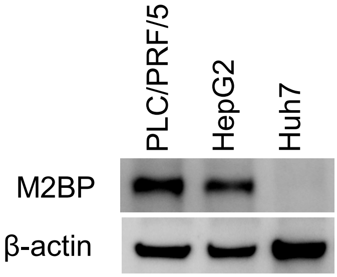

Supplementary Figure 3

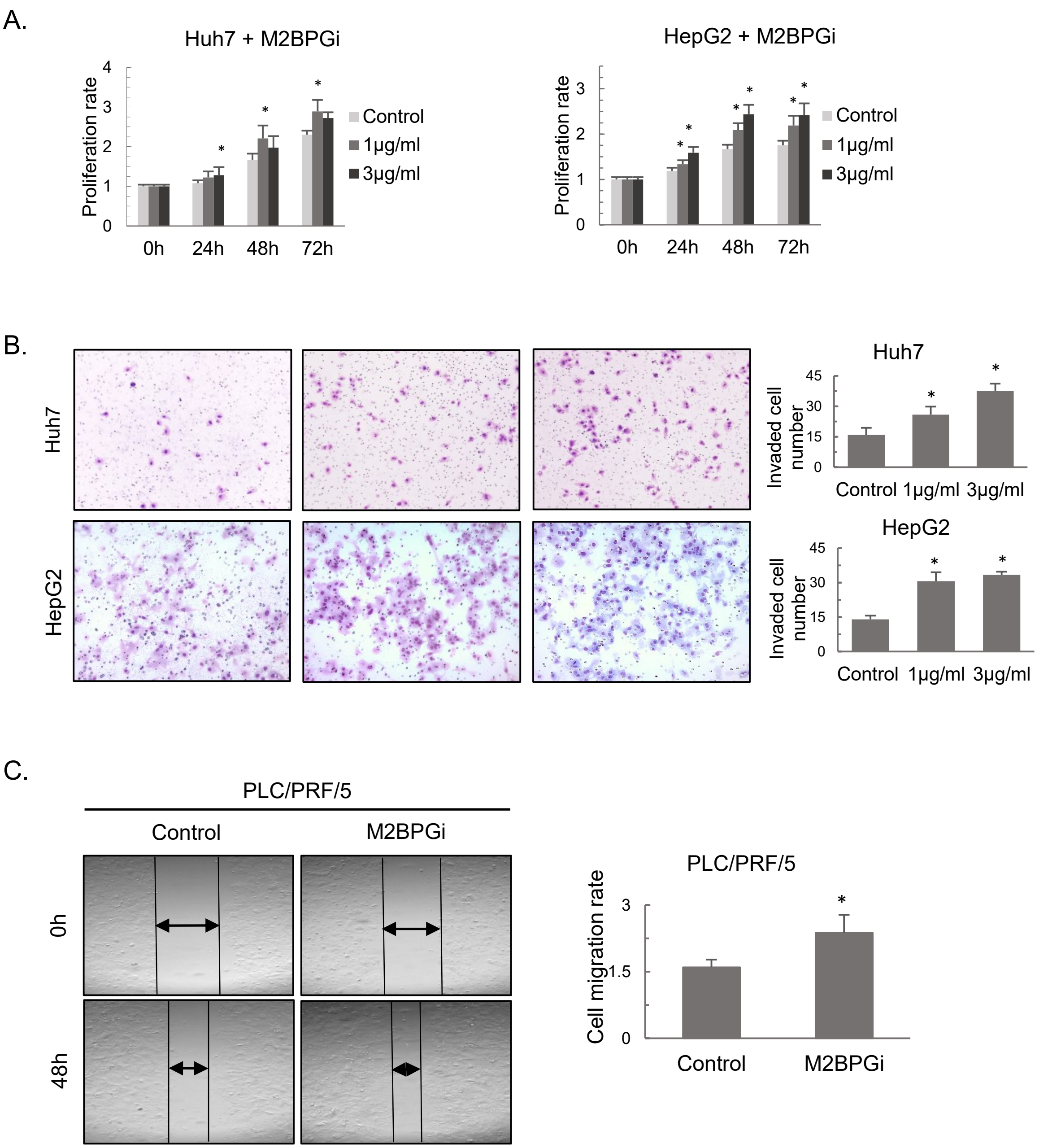

Supplementary Figure 4

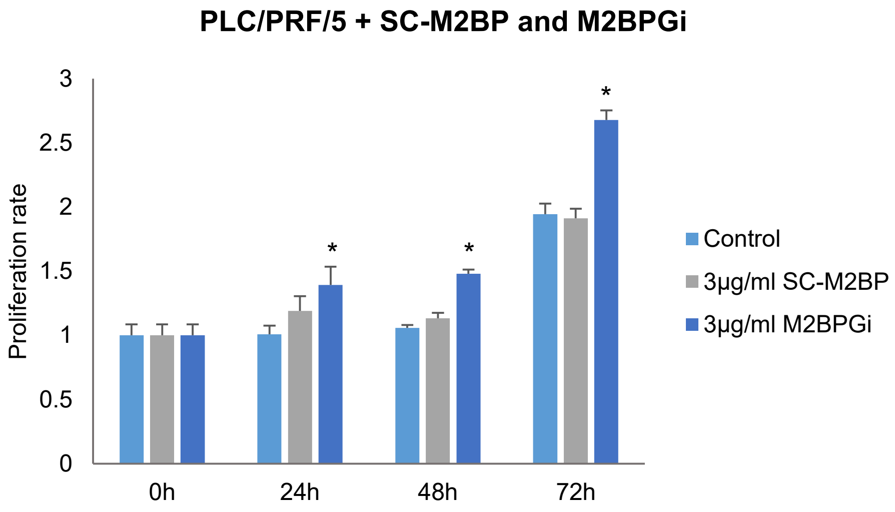

Supplementary Figure 5

A.

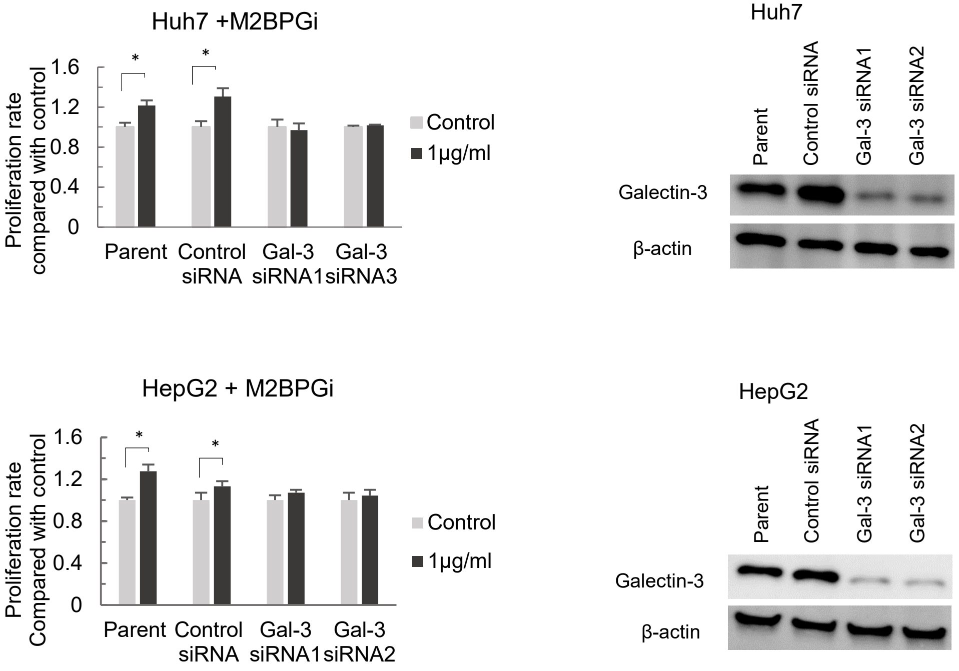

Supplement: Supplementary file 1 — Supplementary Figures [file 41416_2020_971_MOESM1_ESM.pdf]
