# Supplementary material for: Mac-2-binding protein glycan isomer enhances the aggressiveness of hepatocellular carcinoma by activating mTOR signaling
Source: Br J Cancer. 2020 Jul 6;123(7):1145–53. doi: 10.1038/s41416-020-0971-y (PMC7525442; doi:10.1038/s41416-020-0971-y)
Supplement: Supplementary file 2 — Supplementary Figure legends [file 41416_2020_971_MOESM2_ESM.docx]

**Supplementary Figure 1.** Co-localization of M2BPGi and CD68-positive cells (Kupffer cells).

Representative multi-immunofluorescence images of cells labeled to indicate M2BP (red; upper left panel), WFA (green; upper middle panel), M2BP with WFA as M2BPGi (merged; upper right panel), CD68 (pink; lower right panel), or M2BPGi and CD68 (merged; lower middle panel) and stained with hematoxylin and eosin (lower right panel) in the adjacent non-tumor areas of resected hepatocellular carcinoma tissue (×400 magnification).

**Supplementary Figure 2.** *M2BP* mRNA expressed in the stroma of HCC.

M2BP protein expression in the HCC cell lysates. M2BP protein were expressed in the PLC/PRF/5 and HepG2, not in the Huh7 cell line.

**Supplementary Figure 3.** M2BPGi treatment enhanced the progression of HCC *in vitro*

HCC cell line (Huh7 and HepG2) treated with M2BPGi were subjected to (A) a proliferation assay (n = 6, *p < 0.05) and (B) invasion assay (n = 5, *p < 0.05). (C) PLC/PRF/5 cells treated with and without (control) M2BPGi and subjected to migration assay. Cell migration rate were presented in graph (n = 5, *p < 0.05).

**Supplementary Figure 4.** Sugar chain cut off M2BPGi (SC-M2BPGi) had no proliferation effect on HCC cell line.

PLC/PRF/5 cell line were treated with without (control) sugar chain cut off M2BPGi (SC-M2BPGi) and M2BPGi and were subjected to proliferation assay (n = 6, *p < 0.05 vs control). 3 µg/ml SC-M2BPGi did not enhance the proliferation of PLC/PRF/5 cell line compared with control. However, 3 µg/ml M2BPGi enhanced the proliferation of PLC/PRF/5 after 24h, 48h and 72h.

**Supplementary Figure 5.** M2BPGi promoted HCC proliferation in presence of galectin-3 (Gal-3)

Compared with control, M2BPGi did not enhance the proliferation of HCC cell lines (Huh7 and HepG2) treated with Gal-3 siRNA (n = 6 *p < 0.01). Western blotting validated the suppression of Gal-3 by specific siRNAs.
